# Comparative Phytochemical Profiling and Phenotypic Senolytic Screening of Botanical Extracts for Oxidative-Stress-Induced Skin-Cell Senescence

**DOI:** 10.3390/antiox15080920

**Published:** 2026-07-24

**Authors:** Somi Park, Ji Eun Lee, Hyeontae Kang, Kyoung-Min Choi, Hee Cheol Kang, Jin Woo Min

**Affiliations:** 1 Green & Biome Customizing Laboratory, GFC Life Science Co., Ltd., Hwaseong 18471, Republic of Korea; 2 Human & Microbiome Communicating Laboratory, GFC Life Science Co., Ltd., Hwaseong 18471, Republic of Korea

**Keywords:** cellular senescence, botanical extracts, flavonoids, senolytics, phytochemical profiling, oxidative stress, skin aging

## Abstract

Cellular senescence contributes to skin aging through the accumulation of senescent cells and the secretion of senescence-associated secretory phenotype (SASP) factors. Although numerous botanical flavonoids have been reported to possess antioxidant and anti-aging properties, the relationship between phytochemical composition, antioxidant capacity, and senolytic activity in complex botanical extracts remains poorly understood. In this study, twenty botanical extracts were systematically compared using a standardized screening platform that integrated total flavonoid quantification, UPLC-Q-TOF-MS phytochemical profiling, antioxidant evaluation, cytotoxicity assessment, and phenotypic senolytic screening in oxidative-stress-induced human foreskin fibroblast (HFF) senescence models. Among the tested extracts, chlorella, water lily, green tea, and rosemary exhibited the most pronounced senolytic-associated activities while maintaining minimal cytotoxicity toward non-senescent fibroblasts. UPLC-Q-TOF-MS analysis revealed that these extracts possessed distinct flavonoid-enriched phytochemical fingerprints despite producing comparable biological responses. Notably, green tea extract exhibited the strongest antioxidant activity, whereas its phytochemical composition differed substantially from those of the other highly active extracts, indicating that antioxidant capacity alone does not predict senolytic efficacy. Collectively, the findings demonstrate that total flavonoid content, antioxidant activity, and senolytic activity are not necessarily directly correlated and highlight the importance of comprehensive phytochemical characterization combined with phenotypic biological screening for identifying botanical resources with senescence-modulating potential. These results provide a practical comparative strategy for discovering multifunctional botanical ingredients applicable to oxidative-stress-associated skin aging.

## 1. Introduction

Skin aging is a multifactorial biological process closely associated with oxidative stress, chronic inflammation, and the progressive accumulation of senescent cells [1,2]. Recent advances in longevity research have identified cellular senescence as a fundamental hallmark of aging and a major contributor to tissue dysfunction through the secretion of senescence-associated secretory phenotype (SASP) factors [3]. Excessive reactive oxygen species (ROS) production induces mitochondrial dysfunction, DNA damage responses, and persistent inflammatory signaling, ultimately promoting cellular senescence and extracellular matrix degradation in skin tissue. Persistent oxidative stress further amplifies SASP-mediated inflammation, thereby accelerating skin aging and functional deterioration [1].

Accordingly, senotherapeutic strategies targeting senescent cells [4,5] have emerged as promising approaches for healthy aging and skin rejuvenation. Senotherapeutics are generally classified into senolytics, which selectively eliminate senescent cells by restoring apoptotic sensitivity, and senomorphics, which suppress SASP-associated dysfunction without necessarily inducing cell death [6]. While senolytics remove damaged senescent cells, excessive clearance may compromise tissue homeostasis, emphasizing the importance of complementary approaches capable of modulating multiple senescence-associated pathways.

Natural flavonoids and polyphenols have attracted considerable attention as candidate senotherapeutics because of their antioxidant, anti-inflammatory, and redox-regulating properties [7]. Representative flavonoids including quercetin, kaempferol, luteolin, rutin, epigallocatechin gallate, baicalin, and hesperetin have been reported to attenuate oxidative stress [8,9,10], regulate inflammatory signaling, and influence senescence-associated phenotypes through modulation of Nrf2, AMPK, NF-κB, and related signaling pathways [11,12,13,14]. Nevertheless, most previous studies have focused on isolated phytochemicals or individual botanical extracts, making direct comparisons among botanical resources difficult because of substantial differences in extraction procedures, phytochemical composition, analytical platforms, and biological evaluation methods.

Unlike purified compounds, crude botanical extracts represent complex phytochemical matrices composed of major flavonoids together with numerous minor flavonoids, phenolic acids, and other bioactive metabolites. Furthermore, accumulating evidence suggests that phytochemical complexity and matrix-dependent interactions within botanical extracts may contribute to biological activities that cannot be fully reproduced by isolated compounds alone [15]. Increasing evidence indicates that interactions among these constituents may generate synergistic or antagonistic biological effects that cannot be predicted from the activities of isolated compounds alone [14,16,17,18]. Consequently, the biological efficacy of botanical extracts cannot be explained solely by the abundance of individual flavonoids or by conventional antioxidant measurements, highlighting the need for integrating comprehensive phytochemical characterization with functional biological evaluation.

Despite growing interest in botanical senotherapeutics, systematic comparative studies evaluating multiple botanical extracts under standardized experimental conditions remain scarce [19]. A standardized comparative screening strategy is therefore essential for identifying botanical resources with genuine senolytic potential and for establishing reliable relationships between phytochemical composition and biological activity [18]. In the absence of such standardized comparisons, however, it remains difficult to determine whether differences in biological activity originate from the botanical source itself or from variations in extraction procedures, phytochemical composition, analytical platforms, and biological evaluation methods [19]. Consequently, the relationship between phytochemical composition, antioxidant capacity, and phenotypic senolytic activity has not been comprehensively established [20].

Therefore, to enable systematic comparison of botanical resources under identical experimental conditions, the present study established a standardized comparative screening platform integrating total flavonoid quantification, UPLC-Q-TOF-MS phytochemical profiling, antioxidant evaluation, cytotoxicity assessment, and phenotypic senolytic screening using oxidative-stress-induced human foreskin fibroblast (HFF) senescence models. By directly comparing twenty botanical extracts under identical analytical and biological conditions, this study aimed to identify promising botanical resources with senolytic potential while evaluating whether phytochemical composition, antioxidant capacity, and senolytic activity are intrinsically associated. This integrated strategy provides a practical framework for the systematic discovery of multifunctional botanical ingredients targeting oxidative-stress-induced skin aging.

## 2. Materials and Methods

### 2.1. Materials

Twenty botanical plants were obtained from a local agricultural market in Korea and used for extraction, phytochemical characterization, and biological evaluation. All obtained botanical extracts as described in Section 2.2. were dissolved in dimethyl sulfoxide (DMSO; Sigma-Aldrich, St. Louis, MO, USA) to prepare stock solutions, which were diluted with the appropriate culture medium immediately before use.

Fisetin, isoquercetin, naringin, kaemferol, naringenin, genistein, quercetin, hesperitin, isorhamnetin, luteolin, and apigenin, and other analytical standards used for UPLC-Q-TOF-MS analysis were purchased from Sigma-Aldrich (St. Louis, MO, USA). LC-MS grade methanol, acetonitrile, and formic acid were purchased from Honeywell International Inc. (Charlotte, NC, USA).

Human foreskin fibroblasts (HFFs) were obtained from the American Type Culture Collection (ATCC, Manassas, VA, USA) and cultured in Dulbecco’s Modified Eagle Medium (DMEM; Gibco, Grand Island, NY, USA) supplemented with 10% fetal bovine serum (FBS; Grand Island, NY, USA) and 1% penicillin–streptomycin (Grand Island, NY, USA).

For antioxidant evaluation, 2,2-diphenyl-1-picrylhydrazyl (DPPH) and L-ascorbic acid were purchased from Sigma-Aldrich. Total flavonoid content was determined using aluminum chloride, sodium nitrite, and sodium hydroxide analytical reagents.

For senescence and biological assays, hydrogen peroxide (H_2_O_2_) was used to induce oxidative-stress-induced cellular senescence. Cell viability was determined using the EZ-Cytox Cell Viability Assay Kit (DoGenBio, Seoul, Republic of Korea). Apoptosis was analyzed using the Muse^®^ Annexin V and Dead Cell Kit (Luminex Corporation, Austin, TX, USA) and quantified with the Guava^®^ Muse^®^ Cell Analyzer (Luminex Corporation). Confocal fluorescence imaging was performed using the Apoptosis/Necrosis Detection Kit (Abcam, Cambridge, UK), including Apopxin Green, CytoCalcein Violet 450, and 7-AAD. Senescence-associated β-galactosidase activity was evaluated using the Senescence β-Galactosidase Staining Kit (Cell Signaling Technology, Danvers, MA, USA).

All other chemicals and reagents were of analytical grade unless otherwise specified.

### 2.2. Preparation of the Botanical Extracts

Twenty botanical materials, including chlorella (*Chlorella vulgaris*), water lily flower (*Nymphaea alba*), black soybean, lotus leaf (*Nelumbo nucifera*), green tea (*Camellia sinensis*), pine needle (*Pinus densiflora*), strawberry calyx (*Fragaria × ananassa*), rosemary (*Rosmarinus officinalis*), *Centella asiatica*, Japanese honey locust (*Gleditsia japonica*), *Asparagus cochinchinensis*, blue lotus (*Nymphaea caerulea*), nipa palm shoot (*Nypa fruticans* Wurmb), butterfly pea flower (*Clitoria ternatea*), *Lespedeza cuneata*, black bean (*Vigna cylindrica* Skeels), Japanese Honeysuckle (*Lonicera japonica*), bird’s-foot trefoil seed (*Lotus corniculatus* var. *japonica*), citron (*Citrus junos*), and black pomegranate (*Punica granatum*), were used for extraction.

Briefly, dried botanical materials (100 g each) were mixed with 1000 mL of 70% (*v*/*v*) aqueous ethanol at a solid-to-solvent ratio of 1:10 (*w*/*v*) in extraction vessels. Extraction was performed at room temperature (25 ± 2 °C) under continuous agitation (100–200 rpm) for 24 h. The extracts were subsequently filtered through Whatman No. 1 filter paper (Whatman International Ltd., Maidstone, UK) to remove insoluble materials. The filtrates were further centrifuged at 4000× *g* for 20 min to eliminate residual particulates and insoluble debris, and the supernatants containing polyphenol-rich fractions were collected.

The recovered supernatants were concentrated under reduced pressure at temperatures below 40 °C using a rotary evaporator to remove ethanol solvent while minimizing degradation of thermolabile phytochemicals. The concentrated extracts were subsequently freeze-dried to obtain powdered botanical extracts, designated as CHL-E, WL-E, BSB-E, NNL-E, GT-E, PNL-E, SC-E, RM-E, CA-E, GJF-E, AC-E, BL-E, PS-E, BPF-E, LC-E, VC-E, LJ-E, CTS-E, CJ-E, and BP-E, respectively. All dried extracts were stored at 4 °C until further analysis and biological evaluation.

### 2.3. Determination of Total Flavonoid Content

The total flavonoid content of the botanical extracts was determined using the aluminum chloride colorimetric assay with slight modifications [21]. Briefly, 125 μL of each extract solution was mixed with 75 μL of sodium nitrate solution, followed by the addition of 150 μL of 10% (*w*/*v*) aluminum chloride solution. The reaction mixture was incubated at room temperature for 5 min. Subsequently, 750 μL of 1 N sodium hydroxide solution was added, and the mixture was further incubated at room temperature for 15 min to allow color development.

The reacted solutions were transferred to a 96-well plate, and absorbance was measured at 492 nm using a microplate reader (BioTek Instruments, Winooski, VT, USA). Quercetin was used as the reference standard to generate a calibration curve using serially diluted standard solutions prepared under identical reaction conditions. The total flavonoid content of each extract was calculated from the calibration curve and expressed as quercetin equivalents (QE). The calibration curve showed good linearity with the regression equation Y=0.01537x+0.00831 and a correlation coefficient ($R^{2}$) of 0.9925.

### 2.4. UPLC-Q-TOF Analysis of Flavonoid Profiles

Qualitative phytochemical profiling of the botanical extracts was performed using an ACQUITY UPLC system coupled with a Xevo G3 QTof mass spectrometer (Waters Corporation, Milford, MA, USA). Prior to analysis, each freeze-dried extract was dissolved in methanol (10 mg/mL) and filtered through a 0.22 μm membrane filter. Chromatographic separation was achieved on an ACQUITY UPLC BEH C18 column (130 Å, 1.7 μm, 2.1 × 50 mm; Waters Corporation). The mobile phase consisted of 0.1% formic acid in water (solvent A) and 0.1% formic acid in methanol (solvent B). Gradient elution was performed as follows: 90:10 (A:B) from 0 to 1 min, linearly changed to 20:80 at 11 min, further changed to 5:95 at 13 min, maintained until 14 min, returned to 90:10 at 14.1 min, and equilibrated until 15 min. The flow rate was 0.3 mL/min, and chromatograms were monitored over a wavelength range of 210–400 nm.

Mass spectrometric analysis was performed using an electrospray ionization (ESI) source operated in the negative ion mode over an *m*/*z* range of 50–1200. Instrument parameters were set as follows: capillary voltage, 2.50 kV; sampling cone voltage, 40 V; source offset, 80 V; source temperature, 120 °C; desolvation temperature, 50 °C; cone gas flow, 0 L/h; and desolvation gas flow, 800 L/h. Leucine enkephalin was continuously infused as the lock-mass reference to ensure mass accuracy throughout data acquisition.

Raw data were acquired using MassLynx™ software (version 4.2; Waters Corporation) and processed with UNIFI™ software (version 3.8; Waters Corporation). Flavonoid compounds were putatively identified by comparing accurate mass, retention time, isotope distribution, ultraviolet spectra, and fragmentation patterns obtained in MSE mode with an in-house flavonoid library implemented in the UNIFI platform. Candidate compounds were assigned based on library matching scores, mass accuracy, and fragmentation agreement. Relative abundances were estimated from extracted ion chromatogram peak intensities, and major flavonoids were comparatively analyzed according to their retention times and signal intensities.

### 2.5. DPPH Radical Scavenging Activity

The antioxidant activity of the botanical extracts was evaluated using a 2,2-diphenyl-1-picrylhydrazyl (DPPH) radical scavenging assay [22]. Briefly, freeze-dried botanical extracts were dissolved in dimethyl sulfoxide (DMSO) at high concentrations and subsequently diluted to the indicated concentrations using distilled water or reaction buffer. Fresh DPPH solution was prepared immediately before use by dissolving DPPH reagent in methanol under light-protected conditions.

For the assay, aliquots of the extract solutions were mixed with DPPH solution in 96-well plates and incubated in the dark at room temperature for 30 min to allow radical scavenging reactions to occur. During the reaction, a reduction in the purple-colored DPPH radical to its non-radical form resulted in a decrease in absorbance intensity.

Following incubation, absorbance was measured at 517 nm using a microplate reader (BioTek Instruments, Winooski, VT, USA). Methanol containing DPPH solution without sample treatment was used as the negative control, and ascorbic acid was used as the positive control. The DPPH radical scavenging activity of each sample was calculated according to the following equation:

$$\mathrm{DPPH}\;\mathrm{radical}\;\mathrm{scavenging}\;\mathrm{activity}(\%)=\left(1-\frac{\mathrm{Absorbance}\;\mathrm{of}\;\mathrm{treated}\;\mathrm{group}}{\mathrm{Absorbance}\;\mathrm{of}\;\mathrm{control}\;\mathrm{group}}\right)\times 100$$

All experiments were independently performed in triplicate, and the results were expressed as mean ± standard deviation (SD). Ascorbic acid was used as a reference antioxidant control in the DPPH assay. Dose-dependent radical scavenging activity was confirmed, and the IC_50_ value of ascorbic acid under the present experimental conditions was calculated as 1.98 μg/mL.

### 2.6. Cell Viability Assay

The cytotoxic effects of the botanical extracts on human foreskin fibroblasts (HFFs) were evaluated using a WST-1 cell viability assay. HFF cells were maintained in Dulbecco’s Modified Eagle Medium (DMEM) supplemented with 10% fetal bovine serum (FBS) and 1% penicillin–streptomycin (P/S) under humidified conditions at 37 °C in a 5% CO_2_ incubator.

For the assay, cells were seeded into 96-well plates at a density of 1 × 10^4^–1 × 10^5^ cells/well and allowed to attach for 24 h. The botanical extracts were dissolved in dimethyl sulfoxide (DMSO) and diluted with culture medium to the indicated concentrations. Cells were subsequently treated with the extracts for 24–72 h. Following treatment, WST-1 reagent was added to each well at a final concentration of 10% (*v*/*v*), and the plates were incubated for an additional 2 h at 37 °C.

Absorbance was measured at 450 nm using a microplate reader (BioTek Instruments, Winooski, VT, USA). Cell viability was calculated according to the following equation:

$$\mathrm{Cell}\;\mathrm{viability}\;(\%)=\left(\frac{\mathrm{Absorbance}\;\mathrm{of}\;\mathrm{treated}\;\mathrm{group}}{\mathrm{Absorbance}\;\mathrm{of}\;\mathrm{control}\;\mathrm{group}}\right)\times 100$$

Untreated cells were used as the control group, and all experiments were independently performed in triplicate.

### 2.7. Establishment of Senescent HFF Cell Model

Human foreskin fibroblast (HFF) cells were cultured in Dulbecco’s Modified Eagle Medium (DMEM) supplemented with 10% fetal bovine serum (FBS) and 1% penicillin–streptomycin (P/S) at 37 °C in a humidified 5% CO_2_ incubator. Cellular senescence was induced using doxorubicin treatment as previously described, with slight modifications [23]. Briefly, HFF cells were seeded into culture plates and treated with 500 nM doxorubicin for 4 h on day 1. Following treatment, the cells were washed with phosphate-buffered saline (PBS), and the culture medium was replaced with fresh complete medium [24]. The cells were subsequently maintained for approximately 7 days to allow stabilization of the senescent phenotype.

Induction of cellular senescence was confirmed by morphological changes, reduced proliferative capacity, and senescence-associated β-galactosidase (SA-β-gal) staining using a Senescence β-Galactosidase Staining Kit (Cell Signaling Technology, Danvers, MA, USA).

### 2.8. Evaluation of Senolytic Activity

#### 2.8.1. Flow Cytometry-Based Senolytic Assay

Senescent HFF cells were treated with botanical extracts for evaluation of senolytic activity. Briefly, freeze-dried botanical extracts were initially dissolved in dimethyl sulfoxide (DMSO) at high concentrations and subsequently diluted in culture medium to a final treatment concentration of 100 μg/mL. The selected treatment concentration was determined based on preliminary cell viability and apoptosis analyses performed in non-senescent HFF cells, in which more than 90% of normal cell viability was maintained without significant induction of apoptosis following treatment with the botanical extracts.

Following treatment for 24 h, DMSO-treated cells were used as the vehicle control. Cells were washed with phosphate-buffered saline (PBS) and detached using trypsin–EDTA solution. Detached cells were collected by centrifugation and resuspended in assay buffer for apoptosis analysis.

Apoptotic cell populations were analyzed using the Guava^®^ Nexin Reagent (Cytek Biosciences, Fremont, CA, USA), which simultaneously stains Annexin V [25,26] and 7-aminoactinomycin D (7-AAD) [27], according to the manufacturer’s instructions. Briefly, harvested cells were incubated with the staining reagent in the dark at room temperature for the recommended staining period and subsequently analyzed using a Guava^®^ easyCyte™ flow cytometer (Cytek Biosciences, Fremont, CA, USA). The proportions of viable, early apoptotic, and late apoptotic cell populations were determined based on fluorescence distribution patterns.

#### 2.8.2. Fluorescence Microscopy Analysis of Senolytic Activity

To visualize apoptosis induction in senescent HFF cells, fluorescence staining was performed using an Apoptosis/Necrosis Assay Kit (blue, green, red; ab176749, Abcam, Cambridge, UK). Senescent HFF cells were seeded onto sterile coverslips placed in 24-well culture plates and treated with botanical extracts for 24 h under the same conditions described above.

Following treatment, cells were stained according to the manufacturer’s instructions using Apopxin Green Indicator for the detection of phosphatidylserine externalization during early apoptosis, 7-aminoactinomycin D (7-AAD) for the detection of membrane-compromised late apoptotic or necrotic cells, and Cytocalcein Violet 450 for viable cell staining. After staining, coverslips were collected and observed using a confocal fluorescence microscope (Leica Microsystems, Wetzlar, Germany) to evaluate apoptosis-associated fluorescence signals in senescent cells.

#### 2.8.3. Quantitative Real-Time PCR Analysis

As a preliminary mechanistic evaluation, quantitative real-time PCR (qRT-PCR) analysis was performed for the representative extract WL-E. Senescent HFF cells were treated with WL-E for 24 h under the same conditions described above [24]. Total RNA was isolated using a NucleoSpin RNA Plus Kit (Macherey-Nagel, Düren, Germany), and complementary DNA (cDNA) was synthesized using amfiRivert cDNA Synthesis Platinum Master Mix (GenDEPOT, Katy, TX, USA) according to the manufacturers’ instructions.

Quantitative real-time PCR was performed using a SensiFAST™ SYBR No-ROX Kit (Bioline, London, UK) with gene-specific primers listed in Table 1. Relative gene expression levels were normalized to β-actin and calculated using the comparative Ct (2^−ΔΔCt^) method as previously described by Livak and Schmittgen [28]. The expression levels of the anti-apoptotic gene MCL-1 and the senescence-associated secretory phenotype (SASP)-related genes MMP-1, MMP-3, IL-8, and CXCL1 were analyzed.

qPCR amplification was performed under the following cycling conditions: initial denaturation at 95 °C for 2 min, followed by 40 cycles of denaturation at 95 °C for 10 s and annealing/extension at 60 °C for 30 s. Melting curve analysis was subsequently conducted from 65 °C to 95 °C with 0.5 °C incremental increases every 5 s to confirm amplification specificity.

### 2.9. Statistical Method

The assays were conducted in triplicate, and all tabulated results were expressed as means ± standard deviation (SD) and were compared using Student’s *t*-test. A *p*-value of less than 0.05 was considered significant.

## 3. Results

### 3.1. Extraction Yields and Total Flavonoid Contents of Botanical Extracts Expressed as Quercetin Equivalents (QE)

The extraction yields and total flavonoid contents of the botanical extracts are presented in Table 2. Considerable variations in flavonoid abundance were observed among the extracts under identical preparation conditions. Total flavonoid contents were quantified using a quercetin standard calibration curve and expressed as quercetin equivalents (QE) based on extract solutions prepared at 100 μg/mL. Among the tested samples, CHL-E, WL-E, GT-E, and RM-E exhibited relatively high flavonoid contents on an equivalent extract weight basis compared with the other botanical extracts. These flavonoid-enriched extracts were subsequently subjected to further comparative phytochemical and biological evaluations to investigate their potential relevance to senescence-modulating activities.

### 3.2. UPLC-Q-TOF-MS Analysis of Flavonoid Profiles

**Figure 1:**
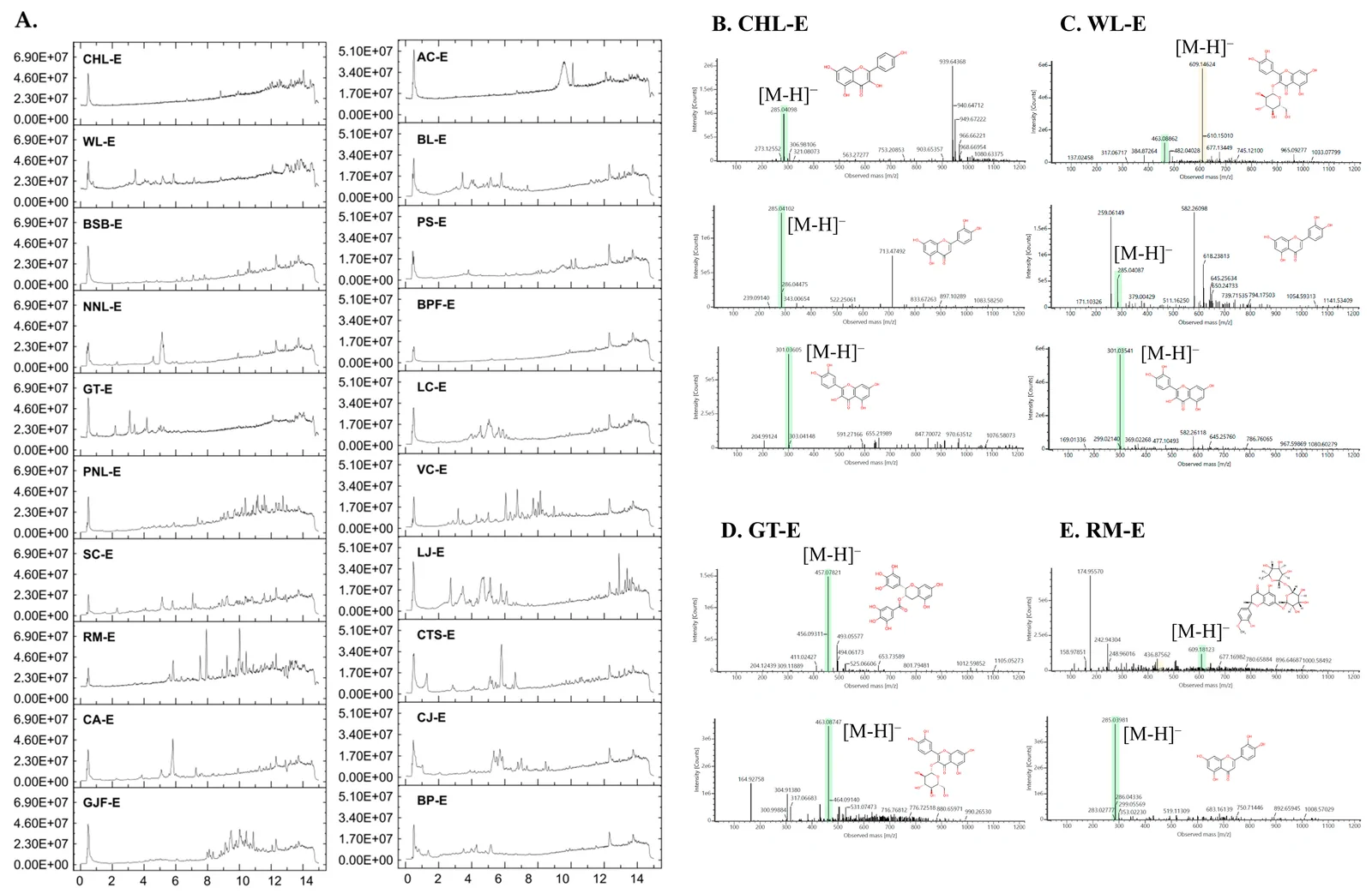
UPLC-Q-TOF-MS characterization of flavonoids in botanical extracts. (**A**) Representative UPLC-Q-TOF-MS base peak chromatograms of the 20 botanical extracts analyzed in this study. Distinct chromatographic profiles were observed among the extracts, reflecting differences in phytochemical composition; (**B**–**E**) Representative MS/MS fragmentation spectra of major flavonoids identified in CHL-E (**B**), WL-E (**C**), GT-E (**D**), and RM-E (**E**). Compound identification was achieved through accurate mass measurements, retention times, isotopic distribution patterns, and characteristic fragmentation ions obtained in negative electrospray ionization (ESI^−^) mode. [M−H]^−^, deprotonated molecular ion.

Comparative UPLC-Q-TOF-MS analysis was performed to characterize the flavonoid composition patterns of the 20 botanical extracts. The detected flavonoid compounds identified from each extract are summarized in Table 3, and representative chromatographic profiles are presented in Figure 1. Across the tested extracts, diverse flavonoid-related compounds were detected with distinct retention times and mass spectral patterns, indicating substantial differences in phytochemical composition among the botanical samples.

Among the analyzed extracts, CHL-E, WL-E, GT-E, and RM-E exhibited relatively enriched and characteristic flavonoid profiles compared with the other botanical extracts. Overall, the major flavonoids detected in CHL-E, WL-E, GT-E, and RM-E were generally consistent with phytochemical constituents previously reported for these botanical sources, although minor differences in compound composition and relative abundance may be attributable to variations in plant origin, extraction procedures, and analytical conditions.

In CHL-E, major flavonoids including baicalin, kaempferol, luteolin, nobiletin, and quercetin were identified with high response intensities. Compared with previously reported metabolomic analyses of *Chlorella* spp., the present analysis identified several flavonoid compounds, suggesting differences in phytochemical composition that may reflect variations in extraction procedures, cultivation conditions, or analytical workflows [29]. The flavonoid composition identified in WL-E, particularly the occurrence of rutin, isoquercetin, quercetin, and kaempferol derivatives, was generally consistent with previous phytochemical analyses of *Nymphaea* species, supporting the reliability of the present UPLC-Q-TOF-MS profiling [30,31]. In particular, rutin and kaempferol displayed relatively high detector responses in WL-E. The catechin-rich phytochemical profile observed for GT-E, particularly the predominance of EGCG-related compounds and procyanidins, was consistent with previous UPLC/LC-MS studies describing the characteristic phenolic composition of green tea (*Camellia sinensis*) extracts [32,33]. RM-E exhibited a characteristic flavonoid profile containing wogonin, luteolin, baicalein, hesperidin, hesperetin, and nobiletin. While luteolin and several flavonoid subclasses have previously been reported in rosemary extracts, the overall phytochemical profile observed in the present study differed from previously reported LC-MS analyses, which may reflect differences in plant materials, extraction procedures, or analytical platforms [34]. Overall, the phytochemical profiles obtained in the present study were generally comparable to previously reported LC-MS analyses, although variations in the occurrence and relative abundance of individual flavonoids were observed, likely owing to differences in plant materials, extraction procedures, and analytical conditions.

In contrast, most of the remaining extracts contained only one or a limited number of identifiable flavonoid compounds. Although nobiletin was detected in several botanical extracts, the overall flavonoid diversity and abundance were considerably lower than those observed in CHL-E, WL-E, GT-E, and RM-E. Furthermore, no major flavonoid compounds were detected in CTS-E, CJ-E, or BP-E under the analytical conditions employed.

Notably, several flavonoids, including quercetin-, kaempferol-, luteolin-, rutin-, and nobiletin-related compounds, were recurrently detected among extracts exhibiting relatively enriched flavonoid abundance. These findings suggested that specific flavonoid composition patterns may contribute to the differential biological activities observed among the botanical extracts and provided a basis for subsequent senescence-modulating activity evaluation.

### 3.3. DPPH Radical Scavenging Activity of Botanical Extracts

**Figure 2:**
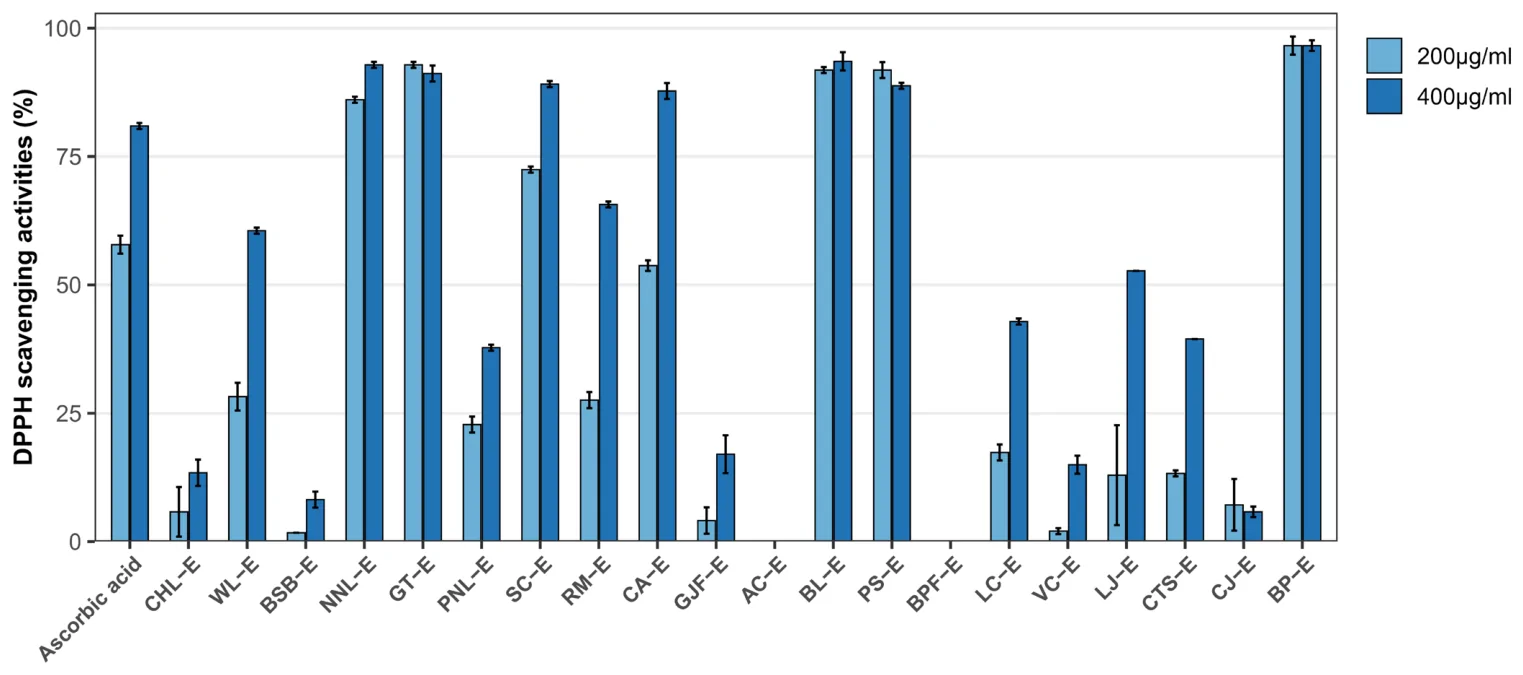
Comparative DPPH radical scavenging activities of 20 botanical extracts. DPPH radical scavenging activities of the botanical extracts were determined at final concentrations of 200 and 400 μg/mL. Radical scavenging activity was expressed as a percentage inhibition of DPPH radicals. Ascorbic acid was included as a positive control. Data are presented as mean ± SD from three independent experiments. Ascorbic acid was used as a positive control (IC_50_ = 1.98 μg/mL).

The antioxidant capacities of the 20 botanical extracts were comparatively evaluated using a DPPH radical scavenging assay (Figure 2). Considerable variations in radical scavenging activity were observed among the tested extracts under identical experimental conditions, suggesting substantial differences in antioxidant potential depending on phytochemical composition. Among the tested samples, CHL-E, WL-E, GT-E, and RM-E exhibited relatively strong antioxidant activities compared with the other botanical extracts.

In particular, GT-E showed the highest DPPH radical scavenging activity among all tested extracts, whereas CHL-E, WL-E, and RM-E also demonstrated comparatively strong free radical-scavenging capacities. These antioxidant activities were generally consistent with the flavonoid-enriched phytochemical profiles identified by UPLC-Q-TOF-MS analysis. CHL-E, WL-E, and RM-E commonly contained flavonoid-related compounds such as quercetin-, kaempferol-, luteolin-, rutin-, and nobiletin-associated structures, which are known to possess antioxidant properties [35,36].

Notably, GT-E exhibited a phytochemical composition pattern distinct from those of the other flavonoid-enriched extracts, being characterized predominantly by catechin-related compounds, including epigallocatechin gallate (EGCG) and procyanidin-associated structures. In particular, catechin-derived polyphenols such as EGCG have been reported to exhibit exceptionally strong antioxidant capacities owing to their multiple hydroxyl groups and gallate moieties, which enhance electron-donating and free radical-scavenging activities [37]. The exceptionally high antioxidant activity of GT-E may therefore be attributed to the strong electron-donating and radical-scavenging capacities of catechin-rich polyphenols containing multiple hydroxyl and gallate moieties. These findings suggest that distinct polyphenolic composition patterns may differentially contribute to the antioxidant potential of botanical extracts.

### 3.4. Effects of Botanical Extracts on Cell Viability in HFF Cells

**Figure 3:**
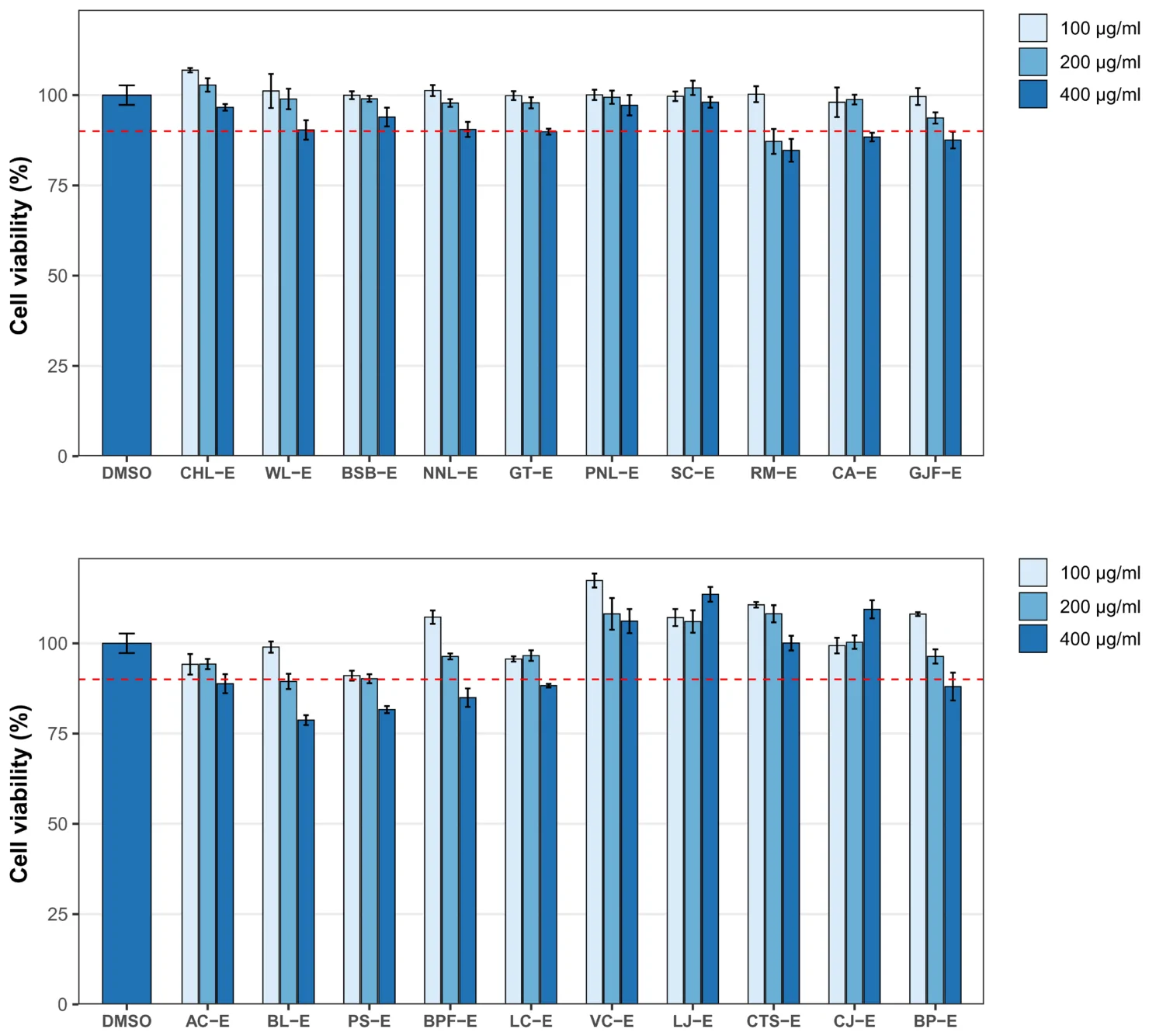
Effects of botanical extracts on the viability of human foreskin fibroblast (HFF) cells. HFF cells were treated with the indicated botanical extracts at final concentrations of 100, 200, and 400 μg/mL for 24 h, and cell viability was evaluated using the WST-1 assay. Cell viability was expressed as a percentage relative to the vehicle-treated control group. The red dashed line indicates the 90% viability threshold used to define the non-cytotoxic concentration range for subsequent biological assays. Data are presented as mean ± SD from three independent experiments. Statistical significance was determined using Student’s *t*-test compared with the vehicle-treated control group.

The cytotoxic effects of the 20 botanical extracts on human foreskin fibroblast (HFF) cells were evaluated using assay following treatment at final concentrations of 100, 200, and 400 μg/mL (Figure 3). Overall, most botanical extracts exhibited minimal cytotoxicity under the tested conditions, maintaining cell viability above 95% even at the highest concentration tested (400 μg/mL). These findings indicated that the majority of the extracts possessed favorable in vitro biocompatibility within the concentration range used for subsequent biological evaluations.

Among the tested samples, several extracts showed moderate reductions in cell viability at higher concentrations. GT-E and RM-E exhibited slight cytotoxicity at 400 μg/mL, resulting in cell viabilities of approximately 89.9% and 87.2%, respectively. BL-E showed reduced cell viability at 200 μg/mL (89.4%), whereas PS-E, BPF-E, and LC-E exhibited comparatively lower cell viabilities at 400 μg/mL, reaching approximately 84.9–88.2%. Nevertheless, none of the tested extracts induced severe cytotoxicity under the experimental conditions.

Based on these results, concentrations used in subsequent senescence-modulating assays were selected within ranges that maintained greater than 90% viability in non-senescent HFF cells. In particular, 100 μg/mL was selected as the primary concentration for comparative senolytic screening to minimize nonspecific cytotoxic effects while allowing evaluation of selective biological activities in senescent cells.

### 3.5. Senolytic Screening of Botanical Extracts in Senescent HFF Cells

**Figure 4:**
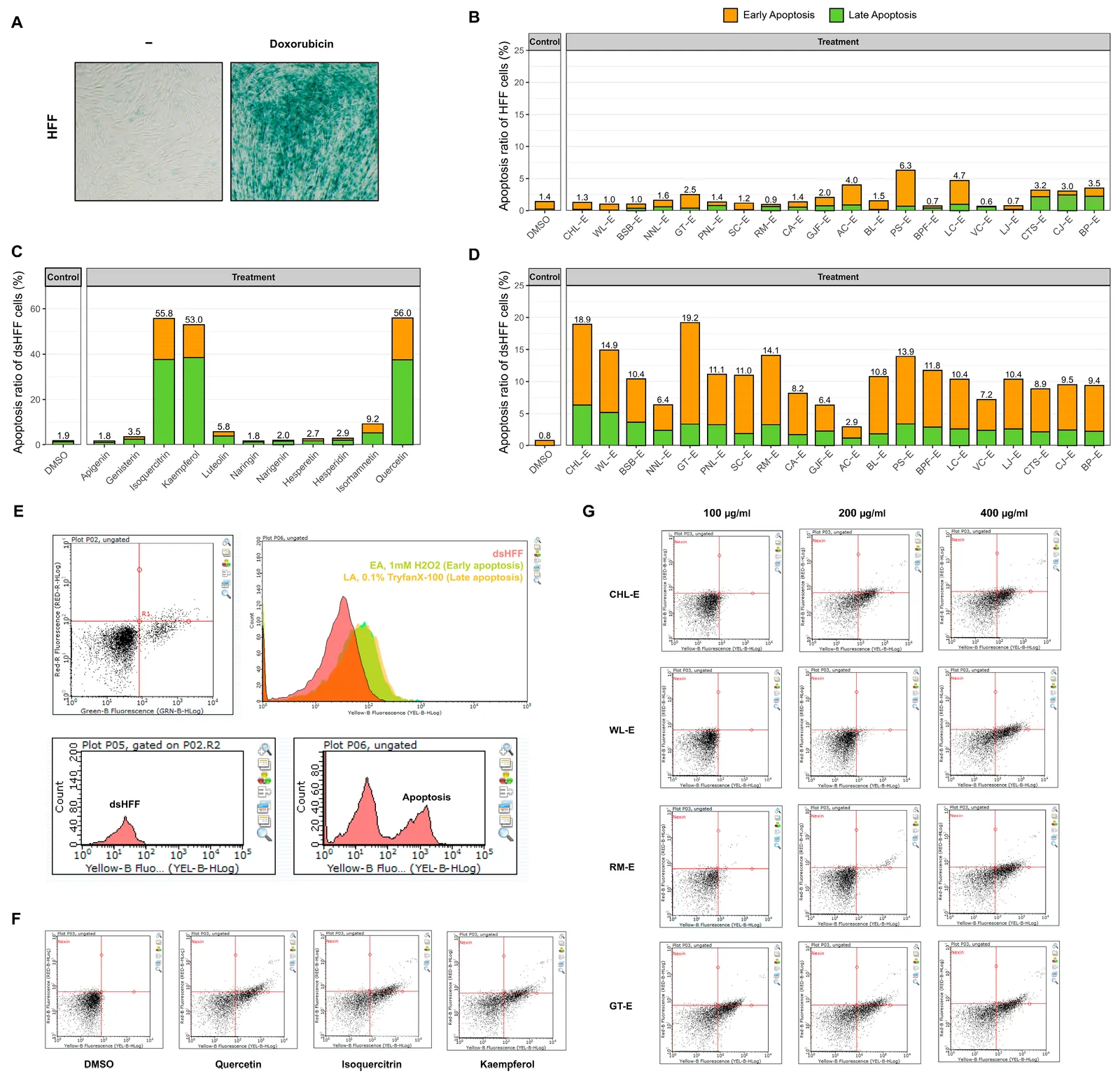
Comparative phenotypic screening of senolytic-associated apoptotic responses induced by botanical extracts and representative flavonoids in doxorubicin-induced senescent human foreskin fibroblasts (dsHFF cells). (**A**) Experimental scheme for dsHFF establishment and apoptosis analysis. (**B**) Apoptotic responses of non-senescent HFF cells following treatment with botanical extracts (100 μg/mL). (**C**) Apoptotic responses of representative flavonoids in dsHFF cells (20 μM). (**D**) Comparative analysis of apoptotic cell populations in dsHFF cells treated with the 20 botanical extracts (100 μg/mL). (**E**) Representative flow cytometric gating strategy and histogram profiles used for apoptosis analysis. (**F**) Representative Annexin V/7-AAD dot plots of dsHFF cells treated with DMSO or representative flavonoids (20 μM). (**G**) Representative Annexin V/7-AAD dot plots of dsHFF cells treated with CHL-E, WL-E, GT-E, and RM-E at 100, 200, and 400 μg/mL.

To evaluate the senolytic potential of the botanical extracts, apoptosis induction in doxorubicin-induced senescent HFF (dsHFF) cells (Figure 4A) was comparatively analyzed using flow cytometry-based Annexin V/7-AAD staining following treatment with the extracts at a final concentration of 100 μg/mL. Under the selected experimental conditions, botanical extract stock solutions prepared in DMSO were diluted into culture medium to minimize nonspecific cytotoxicity while enabling comparative assessment of selective apoptotic responses in senescent cells. To distinguish senolytic activity from nonspecific cytotoxicity, apoptotic responses were first evaluated in non-senescent HFF cells under the same treatment conditions (Figure 4B). Most botanical extracts did not induce significant apoptosis in normal HFF cells at the tested concentration, indicating that the observed apoptotic effects were not primarily caused by general cytotoxicity.

Among the 20 botanical extracts tested, four extracts—CHL-E, WL-E, GT-E, and RM-E—showed markedly enhanced apoptotic induction in dsHFF cells compared with the vehicle-treated control group (Figure 4D). In particular, these extracts induced pronounced increases in early apoptotic cell populations, indicating selective elimination of senescent cells rather than nonspecific necrotic cell death. By contrast, most of the remaining botanical extracts produced minimal changes in apoptotic cell distribution patterns under identical treatment conditions. Representative flavonoids, including quercetin, kaempferol, and isoquercitrin, also promoted early apoptotic responses in dsHFF cells under identical experimental conditions (Figure 4C). The observation that these well-characterized flavonoids exhibited senolytic-associated phenotypes comparable to those of the active botanical extracts further supported the robustness and biological relevance of the screening platform.

Senescent cells are characterized by enhanced resistance to apoptosis through activation of senescence-associated anti-apoptotic pathways (SCAPs) [38]. Therefore, induction of apoptosis in senescent dsHFF cells has been widely used as a phenotypic indicator of senolytic activity. Among the tested botanical extracts, CHL-E, WL-E, GT-E, and RM-E consistently induced greater apoptotic responses than the remaining extracts. Notably, these active extracts also exhibited relatively enriched flavonoid and polyphenolic composition patterns in the UPLC-Q-TOF-MS analysis.

**Figure 5:**
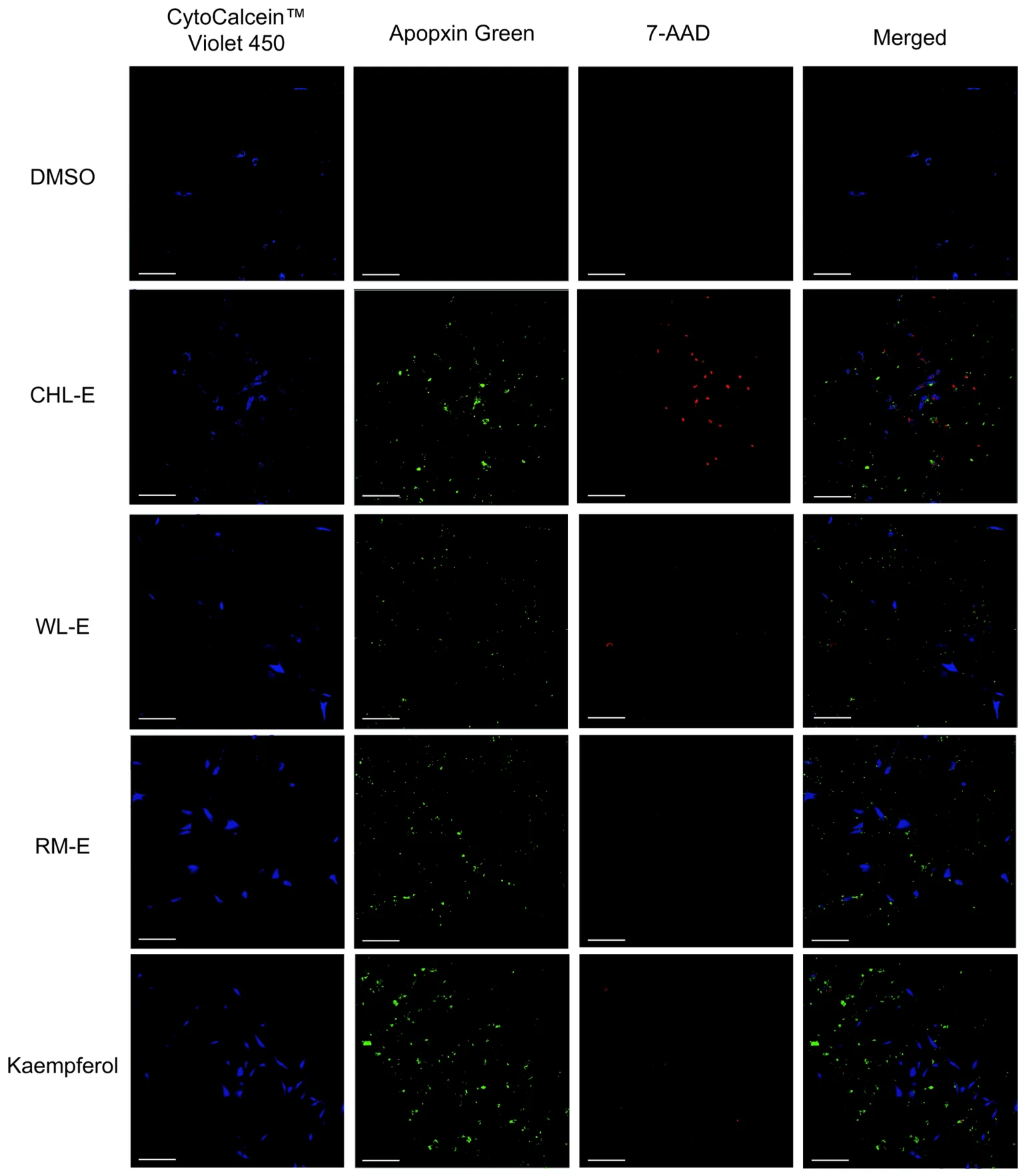
Confocal fluorescence imaging of apoptosis induction in senescent HFF cells following treatment with selected botanical extracts. Representative confocal fluorescence images of doxorubicin-induced senescent HFF (dsHFF) cells treated with vehicle control (1% DMSO), botanical extracts (CHL-E, WL-E, and RM-E; 100 μg/mL), or kaempferol (20 μM) for 24 h. Apoptotic and necrotic cell populations were visualized using the Apoptosis/Necrosis Assay Kit (Abcam, Cambridge, UK), including Cytocalcein Violet 450 (blue; viable cells), Apopxin Green Indicator (green; phosphatidylserine-exposed apoptotic cells), and 7-AAD (red; late apoptotic or necrotic cells). Individual fluorescence channels and merged images are shown for each treatment group. Increased Apopxin Green- and 7-AAD-positive signals were observed in botanical extract-treated groups compared with the vehicle control, indicating enhanced apoptotic responses in senescent cells. Images are representative of three independent experiments. Scale bar = 200 μm.

To further visualize the senolytic-associated effects of the selected botanical extracts, representative samples exhibiting relatively strong apoptotic induction were additionally analyzed by fluorescence imaging using confocal microscopy following treatment with the extracts at a final concentration of 100 μg/mL (Figure 5). Senescent dsHFF cells treated with CHL-E, WL-E, and RM-E showed markedly increased apoptotic fluorescence signals compared with the vehicle-treated control group. In particular, increased Apopxin Green-positive and 7-AAD-positive cell populations were observed following treatment with the flavonoid-enriched extracts, whereas control cells predominantly retained Cytocalcein Violet-positive viable cell signals.

The fluorescence imaging results were generally consistent with the flow cytometry-based apoptosis analysis, supporting the selective apoptotic induction effects of the botanical extracts in senescent cells. Although the proportion of apoptotic cells observed by confocal microscopy appeared lower than that measured by flow cytometry, this difference was expected because the two techniques serve complementary purposes. Flow cytometry quantitatively analyzes apoptosis across the entire cell population, whereas confocal microscopy provides representative visual confirmation within selected microscopic fields [26,39]. Consequently, the fluorescence images were intended to illustrate the cellular localization and morphology of apoptotic responses rather than to provide quantitative estimates of apoptotic cell frequency. Nevertheless, the confocal imaging findings were fully consistent with the flow cytometric results and visually supported the senolytic-associated phenotypic responses induced by the selected botanical extracts.

### 3.6. Preliminary Transcriptional Responses of WL-E in Senescent Dermal Fibroblasts

To obtain preliminary mechanistic insight into the senescence-modulating activity of the selected botanical extracts, quantitative real-time PCR (qRT-PCR) analysis was performed for WL-E, which consistently exhibited strong phenotypic senolytic activity throughout the screening assays. Because senescent cells maintain apoptosis resistance through activation of senescence-associated anti-apoptotic pathways (SCAPs), in which MCL-1 functions as an important pro-survival factor, the expression of MCL-1 was evaluated as a representative molecular marker of anti-apoptotic signaling [38,40]. As shown in Figure S1, WL-E significantly reduced the expression of the anti-apoptotic gene MCL-1 compared with the senescent control. In addition, WL-E markedly suppressed the expression of representative senescence-associated secretory phenotype (SASP)-associated genes, including MMP-1, MMP-3, IL-8, and CXCL1, suggesting coordinated attenuation of pro-inflammatory and extracellular matrix-degrading responses associated with cellular senescence [41].

Collectively, these preliminary findings suggest that WL-E may modulate both senolytic- and senomorphic-associated responses through suppression of anti-apoptotic and SASP-related gene expression.

## 4. Discussion

The present study systematically evaluated the phytochemical characteristics and senolytic potential of twenty botanical extracts using a unified experimental platform integrating total flavonoid quantification, UPLC-Q-TOF-MS profiling, antioxidant activity, cytotoxicity assessment, and phenotypic senolytic screening. Unlike previous studies that primarily focused on the biological activities of individual flavonoids or single botanical sources, the present study directly compared multiple botanical extracts under identical extraction procedures, analytical conditions, and biological assays. This comparative approach enabled the identification of botanical extracts with superior senolytic potential while minimizing experimental variation arising from differences in extraction methods and experimental design.

One of the most notable findings of this study is that total flavonoid content, antioxidant activity, and senolytic activity did not exhibit a direct linear relationship. Although CHL-E, WL-E, GT-E, and RM-E contained relatively high levels of flavonoids and demonstrated superior biological activities compared with the remaining extracts, their individual activity profiles were clearly distinct. In particular, GT-E exhibited the strongest DPPH radical scavenging activity despite possessing a phytochemical composition substantially different from those of CHL-E, WL-E, and RM-E. Likewise, botanical extracts with relatively high total flavonoid contents did not necessarily induce the strongest apoptotic responses in senescent fibroblasts. These findings indicate that antioxidant capacity alone is insufficient to predict senolytic efficacy and suggest that antioxidant and senolytic activities are regulated through distinct biological mechanisms [1,19]. The present results suggest a limited correlation between total flavonoid content and antioxidant or senolytic activity, underscoring the importance of comprehensive phytochemical profiling in the biological evaluation of botanical extracts. Although the DPPH assay is widely used as a rapid and reproducible chemical method for comparing radical-scavenging activity, it primarily reflects direct free-radical scavenging capacity under cell-free conditions and does not fully represent the complexity of intracellular redox regulation or antioxidant defense systems in vivo. In the present study, the DPPH assay was intentionally employed as an initial comparative screening tool to evaluate antioxidant capacity under standardized experimental conditions across twenty botanical extracts. Therefore, the DPPH assay was employed as a standardized comparative screening tool rather than as a comprehensive evaluation of antioxidant activity. Future studies incorporating complementary antioxidant assays (e.g., ABTS, ORAC, and cell-based ROS assays) would provide a more comprehensive characterization of the antioxidant properties of the selected botanical extracts.

Previous studies have reported that many of the identified flavonoids possess potent antioxidant, anti-inflammatory, and senescence-regulating activities. Quercetin is one of the best-characterized natural senolytic flavonoids and has been shown to selectively eliminate senescent cells while suppressing SASP factors [38]. Kaempferol and luteolin have been reported to alleviate oxidative-stress-induced cellular senescence by regulating ROS production and inflammatory signaling pathways [42,43,44]. Rutin and isoquercetin exhibit strong antioxidant activities and enhance cellular resistance to oxidative stress [45,46]. Nobiletin has been reported to protect against oxidative-stress-induced cellular dysfunction and age-associated inflammation through modulation of mitochondrial function and inflammatory signaling [47]. Baicalin, baicalein, wogonin, and epigallocatechin gallate (EGCG) have likewise been reported to attenuate cellular senescence or SASP-associated inflammatory signaling in various experimental models [48,49,50].

However, crude botanical extracts represent highly complex phytochemical matrices composed not only of major flavonoids but also numerous minor flavonoids, phenolic acids, and other bioactive metabolites. Increasing evidence suggests that interactions among these constituents may generate synergistic or antagonistic biological effects that cannot be predicted from the activities of isolated compounds alone [17,18,51]. Indeed, combinations of flavonoids frequently exhibit non-additive antioxidant activities, indicating that the biological properties of crude botanical extracts cannot simply be explained by the abundance of a single dominant phytochemical [51]. Consequently, the biological efficacy of botanical extracts should be interpreted as the integrated outcome of their overall phytochemical composition rather than the concentration of any individual flavonoid.

Previous studies have further demonstrated that interactions among flavonoids are highly dependent on their structural composition and relative abundance [52]. For example, combinations containing epigallocatechin gallate (EGCG) have been reported to exhibit enhanced antioxidant activity through synergistic interactions [37], whereas other flavonoid mixtures display antagonistic effects despite containing potent individual antioxidants. Likewise, the antioxidant activity of quercetin has been shown to increase in the presence of chlorogenic acid, particularly under conditions involving metal and hydrogen ions [53], highlighting the importance of the surrounding phytochemical matrix. These observations indicate that the antioxidant capacity of crude botanical extracts is governed not only by the abundance of individual flavonoids but also by cooperative interactions among coexisting phytochemicals [54]. This concept may explain why GT-E exhibited the strongest DPPH radical scavenging activity despite possessing a phytochemical composition distinct from those of the other highly active botanical extracts.

The results of the present study strongly support this concept. Although CHL-E, WL-E, GT-E, and RM-E exhibited clearly distinct flavonoid fingerprints by UPLC-Q-TOF-MS analysis (Table 3), all four extracts consistently induced greater apoptotic responses in senescent fibroblasts than the remaining botanical extracts (Figure 4 and Figure 5). CHL-E was characterized by baicalin, quercetin, kaempferol, luteolin, and nobiletin, whereas WL-E contained abundant rutin and isoquercetin together with other quercetin derivatives. In contrast, GT-E was dominated by epigallocatechin gallate derivatives and procyanidin C1, while RM-E exhibited a characteristic profile enriched in wogonin, baicalein, hesperidin, and hesperetin. Despite these compositional differences, each extract displayed comparable senolytic-associated activity, suggesting that distinct phytochemical combinations may converge on similar biological phenotypes through different molecular mechanisms. Collectively, these observations support the hypothesis that the senolytic efficacy of crude botanical extracts arises from cooperative interactions among multiple phytochemicals rather than from the activity of a single flavonoid component.

Importantly, the selected botanical extracts selectively promoted apoptosis in senescent fibroblasts while exhibiting minimal cytotoxicity toward non-senescent fibroblasts under identical treatment conditions. Such selective elimination of senescent cells represents a defining characteristic of senolytic agents, which preferentially target cells that have acquired apoptosis resistance through activation of senescence-associated pro-survival pathways (SCAPs) [38]. The consistent findings obtained from flow cytometric apoptosis analysis, together with confocal fluorescence imaging, provide phenotypic evidence consistent with senolytic activity supporting the senolytic potential of the identified botanical extracts.

As an initial effort to explore the molecular basis underlying these phenotypic responses, a preliminary qRT-PCR analysis was performed for WL-E, one of the most active botanical extracts identified during the comparative screening. WL-E reduced the expression of the anti-apoptotic gene MCL-1 (Figure S1) together with representative SASP-associated genes, including MMP-1, MMP-3, IL-8, and CXCL1 (Figure S2). These preliminary observations suggest that WL-E may influence both apoptosis resistance and SASP-associated inflammatory signaling [38,41]. Specifically, MCL-1 expression is presented in Figure S1, whereas representative SASP-associated genes (MMP-1, MMP-3, IL-8, and CXCL1) are shown in Figure S2. However, because this transcriptional evaluation was limited to a single representative extract and was not supported by complementary protein-level validation, these findings should be regarded as exploratory rather than definitive mechanistic evidence. Although increased apoptosis was consistently observed in senescent fibroblasts, the present study did not determine whether cell death occurred through caspase-dependent apoptosis or alternative regulated cell death pathways. Therefore, further mechanistic investigations, including caspase activation assays, mitochondrial apoptotic signaling, and alternative regulated cell death pathways, will be required to clarify the molecular basis of the observed senolytic activity.

The present study employed a doxorubicin-induced senescence model because it is a well-established, reproducible, and widely used in vitro model for inducing stable senescence in dermal fibroblasts. Nevertheless, skin aging is a multifactorial biological process involving chronic oxidative stress, ultraviolet irradiation, inflammation, and extracellular matrix remodeling. Therefore, although the doxorubicin-induced model is suitable for comparative phenotypic screening, it does not fully recapitulate the complexity of physiological skin aging. Future validation using oxidative-stress-induced (H_2_O_2_) and UV-induced senescence models will further strengthen the translational relevance of the identified botanical extracts.

The primary objective of the present study was to identify promising botanical candidates through comparative phytochemical and phenotypic screening rather than to establish their precise molecular mechanisms of action. Therefore, future studies incorporating detailed apoptosis-related signaling analyses (including caspase activation and mitochondrial apoptotic pathways), parallel viability assays in both proliferating and senescent cells to determine the Senolytic Index (SI) and evaluate the selectivity of the identified botanical extracts, protein-level validation, transcriptomic and metabolomic approaches, and in vivo skin-aging models will be necessary to further elucidate the molecular mechanisms underlying the observed senolytic activity and to clarify the contribution of individual phytochemicals and their interactions. Nevertheless, the present work provides one of the few systematic comparative analyses linking phytochemical composition, antioxidant capacity, and phenotypic senolytic activity across a diverse collection of botanical extracts under standardized experimental conditions. More importantly, the findings demonstrate that total flavonoid content, antioxidant activity, and senolytic activity should not be regarded as interchangeable indicators of biological efficacy. Instead, comprehensive phytochemical profiling combined with phenotypic biological screening represents a more reliable strategy for identifying botanical resources with senotherapeutic potential. From a translational perspective, our results further suggest that standardized crude botanical extracts, which preserve the natural complexity of plant-derived phytochemicals, may serve as promising candidates for the development of next-generation cosmetic ingredients targeting oxidative-stress-induced cellular senescence.

## 5. Conclusions

This study provides a systematic comparative evaluation of twenty botanical extracts by integrating phytochemical characterization, antioxidant assessment, cytotoxicity testing, and phenotypic senolytic screening under standardized experimental conditions. Among the tested extracts, CHL-E, WL-E, GT-E, and RM-E consistently exhibited superior senolytic-associated activity while maintaining minimal cytotoxicity toward non-senescent fibroblasts, identifying them as promising botanical candidates for targeting oxidative-stress-induced cellular senescence.

Importantly, our findings demonstrate that total flavonoid content, antioxidant activity, and senolytic activity are not directly correlated, indicating that conventional antioxidant evaluation alone is insufficient to predict senolytic potential. Instead, the biological efficacy of botanical extracts appears to depend on the integrated effects of their complex phytochemical composition rather than on the abundance of individual flavonoids. These observations highlight the importance of combining comprehensive phytochemical profiling with functional biological screening for the discovery of senotherapeutic botanical resources.

Preliminary transcriptional analysis of WL-E further suggested modulation of apoptosis- and SASP-associated genes, providing initial mechanistic insight into its senescence-modulating activity. Although these exploratory findings require additional validation through protein-level and in vivo studies, they support the potential of selected botanical extracts to influence multiple senescence-related pathways.

Overall, this work establishes a practical comparative screening strategy for identifying senolytic botanical extracts and provides valuable phytochemical and biological evidence supporting the development of standardized crude botanical extracts as next-generation cosmetic ingredients for mitigating oxidative-stress-induced skin aging.

Taken together, the present study provides a foundation for future investigations linking phytochemical complexity with senotherapeutic efficacy and supports the rational development of multifunctional botanical ingredients for skin-aging intervention.

## Figures and Tables

**Table 1:** Primer sequences used for quantitative real-time PCR analysis.

Target Gene	Gene Accession Number	Sequences
hBeta-actin (F)	NM_001101.3	5′-ACTCTTCCAGCCTTCCCTCC-3′
hBeta-actin (R)		5′-CGTACAGGTCTTTGCGGATG-3′
hMCL-1 (F)	NM_021960.5	5′-CCAAGAAAGCTGCATCGAACCAT-3′
hMCL-1 (R)		5′-CAGCACATTCCTGATGCCACCT-3′
hMMP-1 (F)	NM_002421.4	5′-CCCAGCGACTCTAGAAACAC-3′
hMMP-1 (R)		5′-GCCTCCCATCATTCTTCAGG-3′
hMMP-3 (F)	NM_002422.5	5′-CACTCACAGACCTGACTCGGTT-3′
hMMP-3 (R)		5′-AAGCAGGATCACAGTTGGCTGG-3′
hIL-8 (F)	NM_000584.4	5′-ACTGAGAGTGATTGAGAGTGGAC-3′
hIL-8 (R)		5′-AACCCTCTGCACCCAGTTTTC-3′
hCXCL-1 (F)	NM_001511.4	5′-AGCTTGCCTCAATCCTGCATCC-3′
hCXCL-1 (R)		5′-TCCTTCAGGAACAGCCACCAGT-3′

**Table 2:** Total flavonoid contents of botanical extracts.

Extract	Botanical Source	Extraction Yield (%)	Total Flavonoid Content (μg QE/mL at 100 μg/mL) ^1^
CHL-E	Chlorella vulgaris	17.9	183.7 ± 1.3
WL-E	Water lily flower	3.1	307.0 ± 5.6
BSB-E	Black soybean	2.8	38.4 ± 4.4
NNL-E	Lotus leaf	5.6	15.6 ± 1.5
GT-E	Green tea	20	103.6 ± 2.7
PNL-E	Pine needle	6.8	46.3 ± 1.2
SC-E	Strawberry calyx	3.4	17.7 ± 3.4
RM-E	Rosemary	15.0	140.9 ± 16.4
CA-E	Centella asiatica	18.8	87.5 ± 12.1
GJF-E	Korean honey locust	7.9	39.2 ± 3.2
AC-E	Asparagus cochinchinensis	13.4	72.0 ± 8.3
BL-E	Blue lotus	2.5	90.3 ± 6.8
PS-E	Nipa palm shoot	8.9	60.8 ± 3.6
BPF-E	Butterfly pea flower	6.7	58.0 ± 1.5
LC-E	Lespedeza cuneata	9.3	4.78 ± 0.2
VC-E	Black bean	4.1	16.5 ± 0.3
LJ-E	Japanese Honeysuckle	11.3	7.9 ± 1.1
CTS-E	Bird’s-foot trefoil seed	10.6	18.2 ± 2.1
CJ-E	Citron	3.2	16.8 ± 2.8
BP-E	Black pomegranate	2.9	4.8 ± 0.8

^1^ Results are reported as the means ± SE of independent experiments performed in triplicate.

**Table 3:** Comparative flavonoid profiles of 20 botanical extracts with tentatively identified compounds characterized by UPLC-Q-TOF-MS.

Extract	Compound	RT (min)	Neutral Mass (Da)	Formula	Observed *m*/*z*
CHL-E	Baicalin	0.48	446.08491	C_21_H_18_O_11_	445.0739
	Kaempferol	7.49	286.04774	C_15_H_10_O_6_	285.0410
	Luteolin	6.92	286.04774	C_15_H_10_O_6_	285.0410
	Nobiletin	12.06	402.13147	C_21_H_22_O_8_	401.1242
	Quercetin	6.66	302.04265	C_15_H_10_O_7_	301.0360
WL-E	Isoquercetin	5.24	464.09548	C_21_H_20_O_12_	463.0886
	Kaempferol	7.47	286.04774	C_15_H_10_O_6_	285.0403
	Luteolin	6.93	286.04774	C_15_H_10_O_6_	285.0409
	Nobiletin	12.08	402.13147	C_21_H_22_O_8_	401.1225
	Quercetin	6.65	302.04265	C_15_H_10_O_7_	301.0354
	Rutin	5.23	610.15338	C_27_H_30_O_16_	609.1462
BSB-E	nobiletin	7.90	402.13147	C_21_H_22_O_8_	401.1273
NNL-E	nobiletin	12.31	402.13147	C_21_H_22_O_8_	401.1279
GT-E	(+)-Epigallocatechin gallate	1.66	458.08491	C_22_H_18_O_11_	457.0782
	Isoquercetin	5.14	464.09548	C_21_H_20_O_12_	463.0875
	Nobiletin	12.11	402.13147	C_21_H_22_O_8_	401.1222
	Procyanidin C1	0.5	866.20581	C_45_H_38_O_18_	865.1996
	Rutin	5.24	610.15338	C_27_H_30_O_16_	609.1448
PNL-E	nobiletin	12.31	402.13147	C_21_H_22_O_8_	401.1281
	Vinpocetine	6.54	350.19943	C_22_H_26_N_2_O_2_	349.1916
SC-E	nobiletin	12.32	402.1315	C_21_H_22_O_8_	401.1281
RM-E	Baicalein	7.60	270.05282	C_21_H_18_O_11_	269.0449
	hesperetin	7.22	302.07904	C_16_H_14_O_6_	301.0712
	hesperidin	5.71	610.18977	C_28_H_34_O_15_	609.1812
	Luteolin	6.94	286.04774	C_15_H_10_O_6_	285.0398
	nobiletin	12.11	402.13147	C_21_H_22_O_8_	401.1223
	Wogonin	9.40	284.06847	C_16_H_12_O_5_	283.0604
CA-E	isoquercetin	5.23	464.09548	C_21_H_20_O_12_	463.0923
	nobiletin	12.31	402.13147	C_21_H_22_O_8_	401.1277
GJF-E	nobiletin	12.31	402.1315	C_21_H_22_O_8_	401.1272
AC-E	Naringenin	6.90	272.06847	C_15_H_12_O_5_	271.0604
	Nobiletin	12.11	402.13147	C_21_H_22_O_8_	401.1220
BL-E	Isorhamnetin	7.15	316.0583	C_16_H_12_O_7_	315.0542
	nobiletin	12.32	402.13147	C_21_H_22_O_8_	401.1276
PS-E	nobiletin	12.32	402.13147	C_21_H_22_O_8_	401.1277
BPF-E	nobiletin	12.32	402.13147	C_21_H_22_O_8_	401.1279
LC-E	N.M.				
VC-E	hesperetin	6.85	302.07904	C_16_H_14_O_6_	301.0741
LJ-E	hesperidin	8.93	610.18977	C_28_H_34_O_15_	609.1800
CTS-E	N.M.				
CJ-E	N.M.				
BP-E	N.M.				

RT, retention time; *m*/*z*, mass-to-charge ratio. Compound assignments were tentatively performed based on accurate mass measurements, molecular formula prediction, and comparison with an in-house flavonoid library and previously reported literature data. N.M., no matching compound identified in the flavonoid library.

## Data Availability

All relevant data is provided within the manuscript.

## Supplementary Materials

The following supporting information can be downloaded at: https://www.mdpi.com/article/10.3390/antiox15080920/s1. Figure S1: Preliminary effect of WL-E on the expression of the anti-apoptotic marker MCL-1 in senescent dsHFF cells; Figure S2: Preliminary effect of WL-E on the expression of senescence-associated secretory phenotype (SASP)-related genes (MMP-1, MMP-3, IL-8, and CXCL1) in senescent dsHFF cells.

## Abbreviations

The following abbreviations are used in this manuscript:

ANOVA	Analysis of variance
β-actin	Beta-actin
BSA	Bovine serum albumin
DMEM	Dulbecco’s Modified Eagle Medium
DMSO	Dimethyl sulfoxide
DPPH	2,2-Diphenyl-1-picrylhydrazyl
dsHFF	Doxorubicin-induced senescent human foreskin fibroblast
EGCG	Epigallocatechin gallate
EIC	Extracted ion chromatogram
FBS	Fetal bovine serum
FSC	Forward scatter
HFF	Human foreskin fibroblast
IL-8	Interleukin-8
MCL-1	Myeloid cell leukemia-1
MMP-1	Matrix metalloproteinase-1
MMP-3	Matrix metalloproteinase-3
*m*/*z*	Mass-to-charge ratio
Nrf2	Nuclear factor erythroid 2-related factor 2
PBS	Phosphate-buffered saline
qRT-PCR	Quantitative real-time polymerase chain reaction
ROS	Reactive oxygen species
RT	Retention time
SASP	Senescence-associated secretory phenotype
SCAPs	Senescence-associated anti-apoptotic pathways
SD	Standard deviation
SSC	Side scatter
TFC	Total flavonoid content
UPLC-Q-TOF-MS	Ultra-performance liquid chromatography–quadrupole time-of-flight mass spectrometry
QE	Quercetin equivalent
