# Supplementary material for: Comparative Phytochemical Profiling and Phenotypic Senolytic Screening of Botanical Extracts for Oxidative-Stress-Induced Skin-Cell Senescence
Source: Antioxidants (Basel). 2026 Jul 24;15(8):920. doi: 10.3390/antiox15080920 (PMC13509742; doi:10.3390/antiox15080920)
Supplement: Supplementary file 1 [file antioxidants-15-00920-s001.zip › antioxidants-4443912-supplementary.pdf]

Supplementary Figures

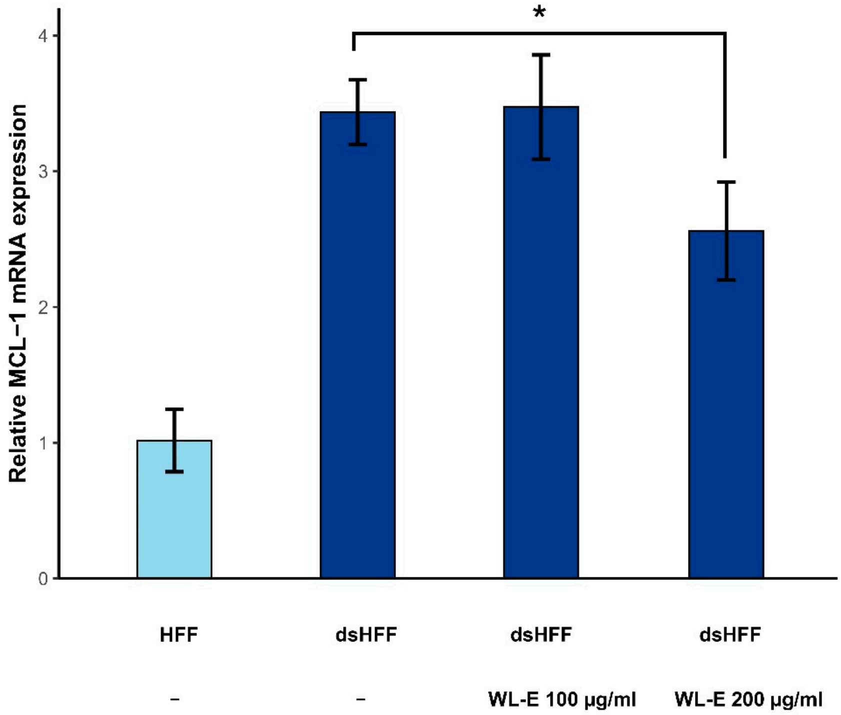

Figure S1. Preliminary effect of WL-E on the expression of the anti-apoptotic marker MCL-1 in doxorubicin-induced senescent human foreskin fibroblasts (dsHFF cells).
Relative mRNA expression of MCL-1 was determined by quantitative real-time PCR following WL-E treatment in oxidative stress-induced senescent human foreskin fibroblasts (dsHFF cells). MCL-1 was analyzed as a representative anti-apoptotic marker associated with senescence-associated anti-apoptotic pathways (SCAPs). Data are presented as the mean ± SD (n = 3). Because this analysis was performed as a preliminary mechanistic evaluation using a single representative extract (WL-E), the results are provided as supplementary information. . **P* < *0.05 vs.* dsHFF*; **P* < *0.01 vs.* dsHFF.

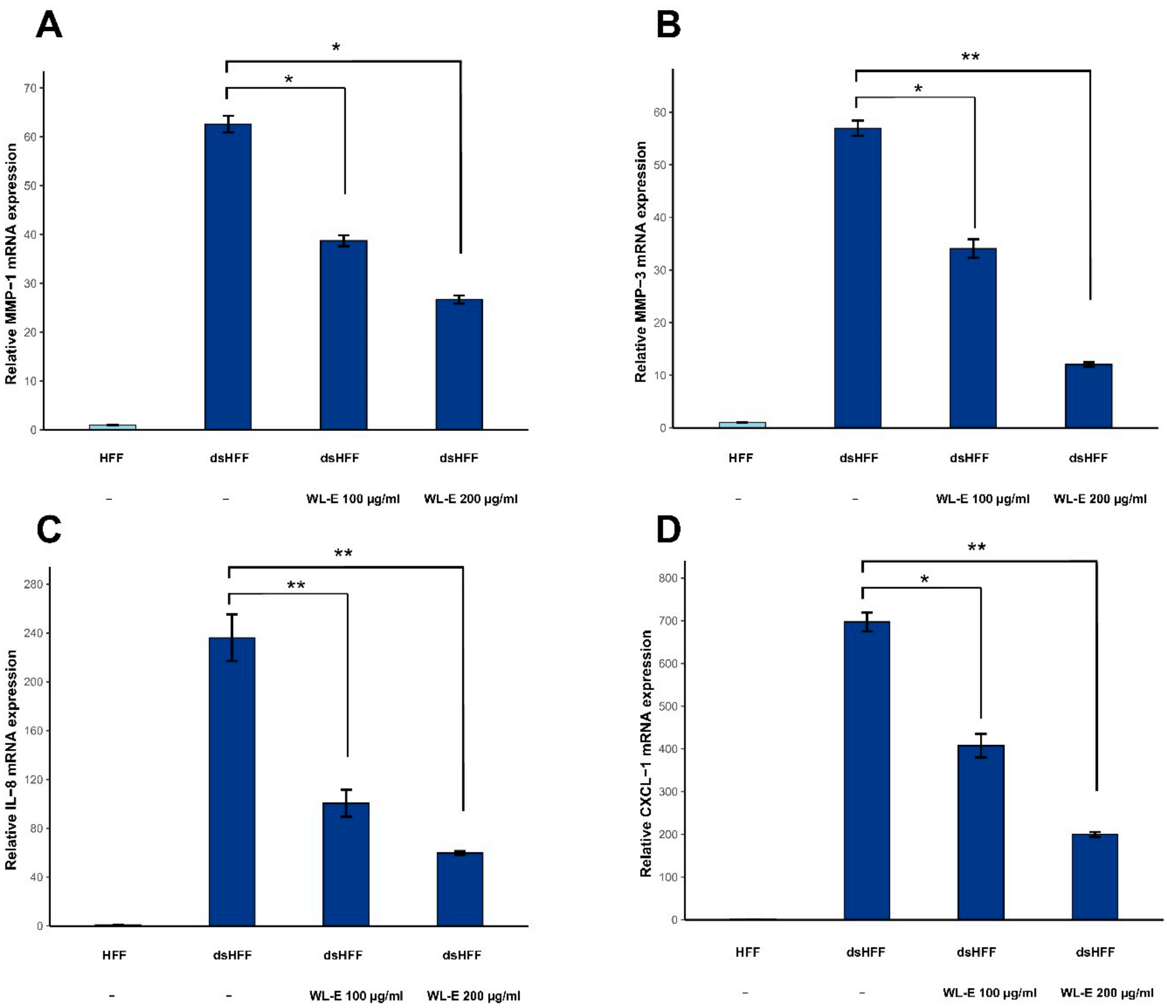

Figure S2. Preliminary effect of WL-E on the expression of senescence-associated secretor y phenotype (SASP)-related genes in doxorubicin-induced senescent human foreskin fibro blasts (dsHFF cells). Relative mRNA expression levels of (A) MMP-1, (B) MMP-3, (C) IL-8, and (D) CXCL-1 were determined by quantitative real-time PCR following WL-E treatment in oxidative stress-induced senescent human foreskin fibroblasts (dsHFF cells). These genes were analyzed as representative SASP-associated markers involved in extracellular matrix remodeling and pro-inflammatory signaling. Data are presented as the mean ± SD (n = 3). Because this analysis was performed as a preliminary mechanistic evaluation using a single representative extract (WL-E), the results are provided as supplementary information. *$P < 0.05$ *vs.* dsHFF; **$P < 0.01$ *vs.* dsHFF.
